# Supplementary material for: Mitigation of Membrane Fouling Using an Electroactive Polyether Sulfone Membrane
Source: Membranes (Basel). 2020 Jan 30;10(2):21. doi: 10.3390/membranes10020021 (PMC7074576; doi:10.3390/membranes10020021)
Supplement: Supplementary file 1 [file membranes-10-00021-s001.pdf]

membranes

Supplementary Materials

Mitigation of membrane fouling using an electroactive polyether sulfone membrane

Chunyan Ma [1,2], Chao Yi [1], Fang Li[1,2], Chensi Shen [1,2], Zhiwei Wang [2,3], Wolfgang Sand[1,4] and Yanbiao Liu [1,2,*]

1 Textile Pollution Controlling Engineering Center of Ministry of Environmental Protection, College of Environmental Science and Engineering, Donghua University, Shanghai 201620, P. R. China; machunyan@dhu.edu.cn (C.M.); 2181557@mail.dhu.edu.cn (C.Y.); lifang@dhu.edu.cn (F.L.); shencs@dhu.edu.cn (C.S.); sand@mikrobiologie.uni-hamburg.de (W.S.)

2 Shanghai Institute of Pollution Control and Ecological Security, Shanghai 200092, P. R. China; zwwang@tongji.edu.cn (Z.W.)

3 State Key Laboratory of Pollution Control and Resource Reuse, School of Environmental Science and Engineering, Tongji University, Shanghai 200092, P. R. China

4 Institute of Biosciences, Freiberg University of Mining and Technology, 09599 Freiberg, Germany

* Correspondence: yanbiaoliu@dhu.edu.cn; Tel.: +86 21 6779 8752

Supplementary Materials: 2 tables and 8 figures.

membranes

MDPI

Materials and methods:

Static protein adsorption experiment.

BSA-FITC solution was used to measure the protein adsorption behavior of Car-PES membranes. The flow channel was filled with staining fluid at room temperature for 1 h in the dark in order to stain the BSA on the membrane. It was then rinsed with 0.5 mg/mL potassium phosphate buffer solution (PBS) to reduce the interference due to background fluorescence. The adsorption was imaged by a fluorescence microscope (ZIESS A1, Carl Zeiss AG, Germany) equipped with an excitation filter of 495 nm and an emission filter of 525 nm.

Filtration velocity method

According to the Guerout-Elford-Ferry equation, mean pore radius D could be experimentally determined by [1]:

$$D = \sqrt{\frac{(2.90-1.75\varepsilon)\times 8\eta\ell Q}{\varepsilon\times A\times\Delta P}} \times 2 \quad (1)$$

where η is the water viscosity (8.9×10^{-4} Pa·s), ℓ is the membrane thickness (nm), Q is the volume of the permeate water per unit time ($m^3 \cdot s^{-1}$), ε is the membrane porosity, A is the membrane effective area (m^2) and ΔP is the operational pressure (0.1 MPa).

Table S1. Properties of the PES and Car-PES membrane.

membrane	electrical conductivity	pore size (nm)	flux of deionized water (LMH)	R_a(nm)
PES	∞	77.0±4.3	421.5±44.6	77.07
Car-PES	5±2.1 Ω·cm	65.1±5.8	374.0±20.1	41.26

Table S2. Properties of the four foulants.

foulant	diameter	Zeta potential (mV)	specific value (mV/nm)
BSA	5.4±0.6 nm	-11.6	2.150
SA	346.2±34.7 nm	-26.5	0.080
yeast	3.3± 0.4 μm	-11.2	0.003
Emulsified oil	13.6±14.5 μm	-56.3	0.004

membranes

MDPI

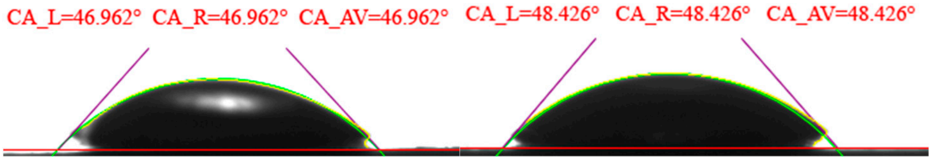

Figure S1. Contact angle images: (**a**) PES membrane with contact angle of 46.9°, (**b**) Car-PES membrane with contact angle of 48.4°.

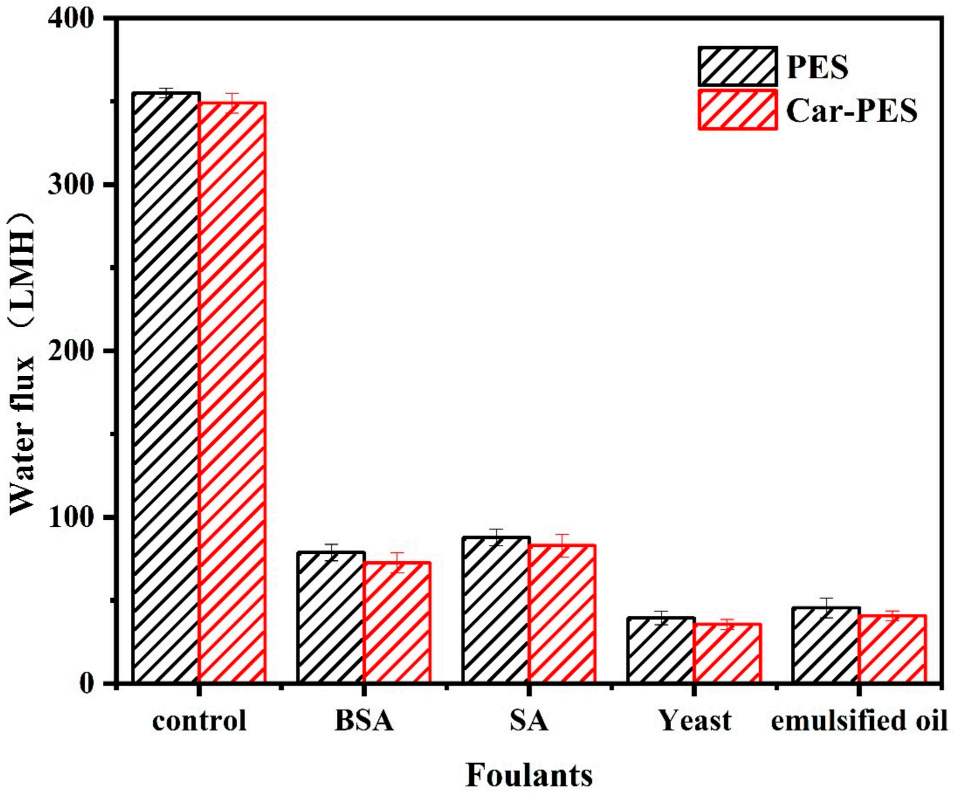

Figure S2. Water fluxes through PES and Car-PES membranes over 24h. (Conditions: $(BSA)_{in}$=10 mg/L, $(SA)_{in}$=10 mg/L, $(yeast)_{in}$=10 mg/L, $(emulsified\ oil)_{in}$=10 mg/L, (cross-flow velocity)=6.1 cm/s, (time)=24 h, and (pressure)= 0.1 bar).

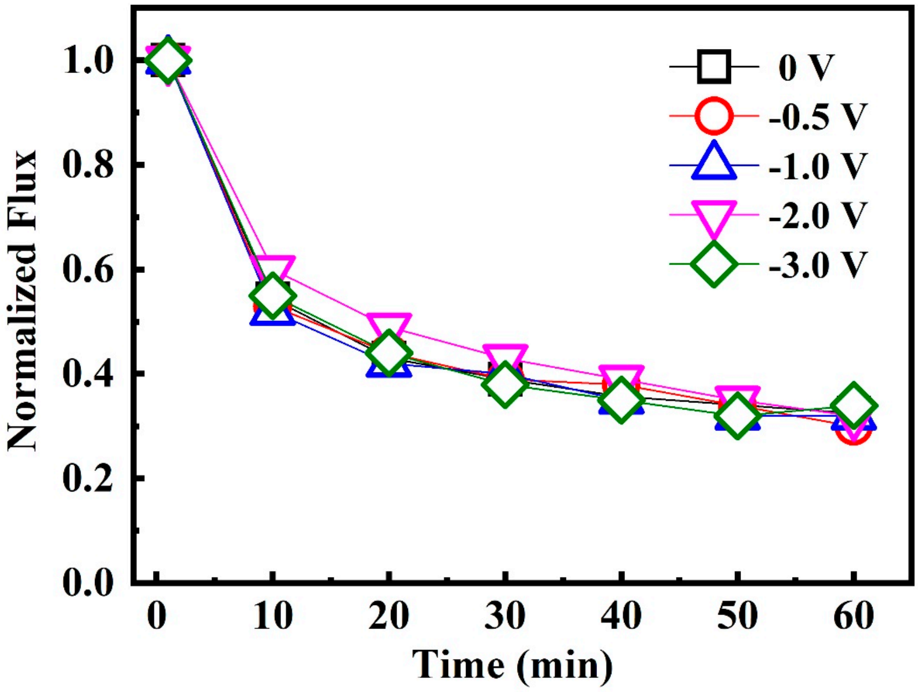

Figure S3. Normalized flux through a PES membrane in comprehensive anti-fouling experiments with different negative voltages applied (Conditions: (SA)in= 10 mg/L, (Na_2SO_4)= 10 mM, (cross-flow velocity)= 6.1 cm/s, and (pressure)= 0.1 bar).

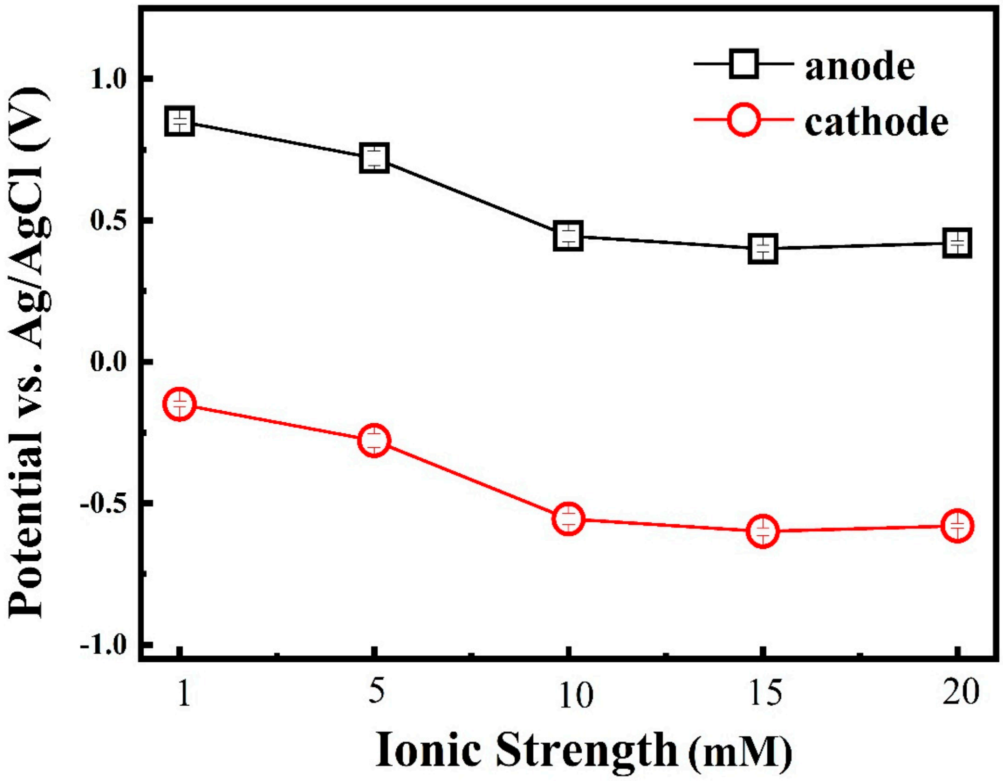

Figure S4. Anode/cathode potential distribution as a function of total cell potential at different ionic strengths: (Conditions: Car-PES memebrane cathode, titanium plate anode, and silver/silver chloride (Ag/AgCl) contrast electrode, $(SA)_{in}$= 10 mg/L).

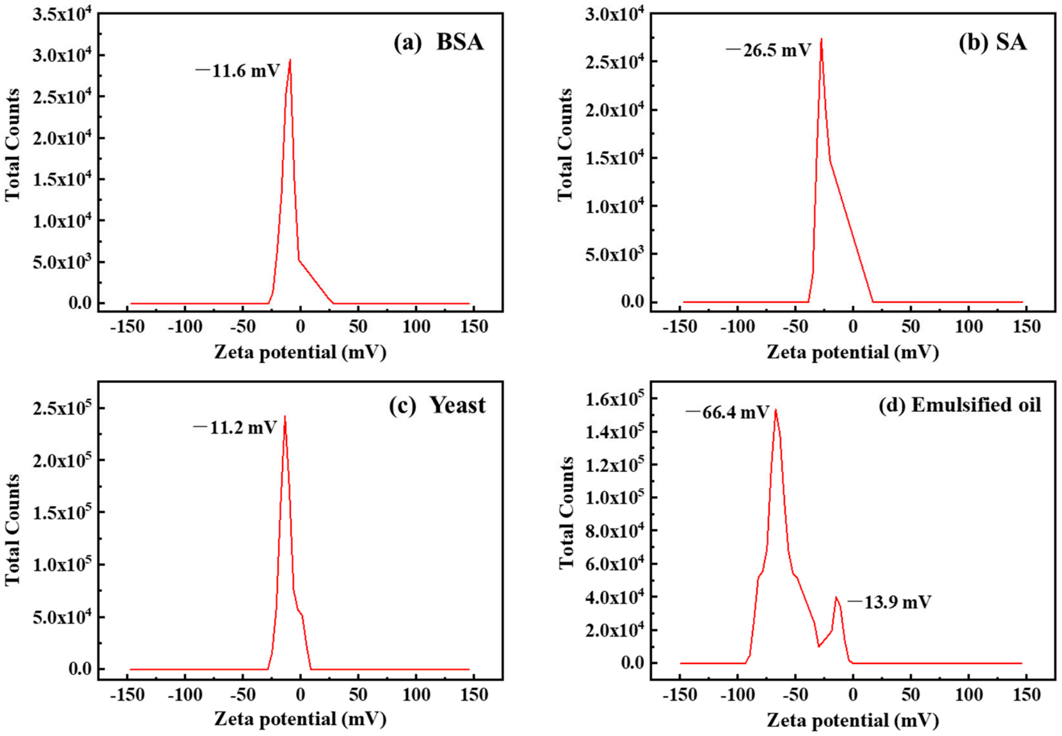

Figure S5. Zeta potential: (**a**) BSA, (**b**) SA, (**c**) yeast, (**d**) emulsified oil. (Conditions: $(BSA)_{in}$= 10 mg/L, $(SA)_{in}$= 10 mg/L, $(yeast)_{in}$= 10 mg/L , $(emulsified\ oil)_{in}$= 10 mg/L, PH= 7.2).

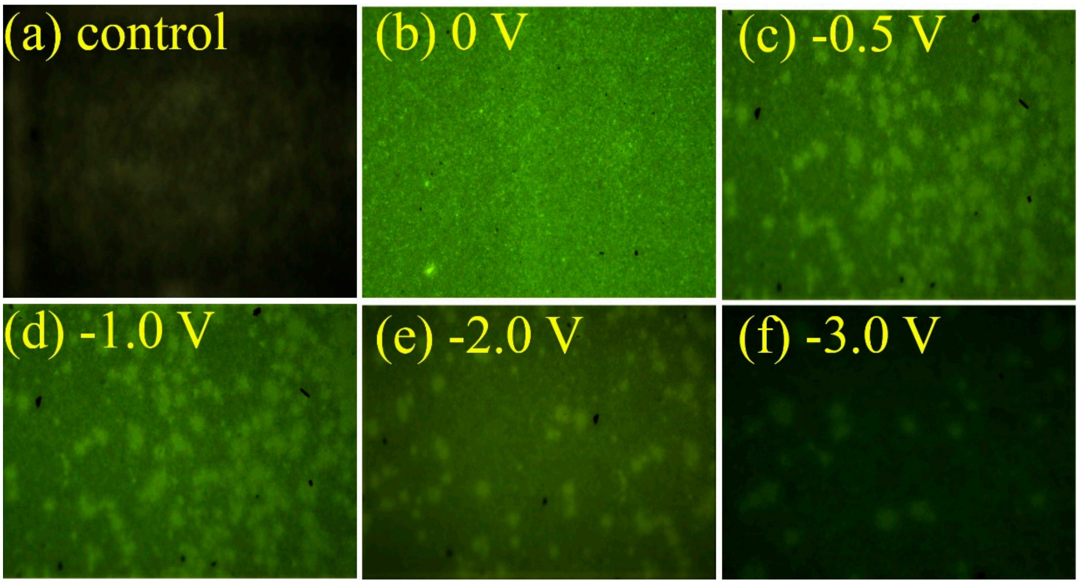

Figure S6. Protein adsorption of at different initial electric fields: (**a**) Control, (**b**) 0 V, (**c**) -0.5 V, (**d**) -1 V, (**e**) -2 V, (**f**) -3 V.

membranes

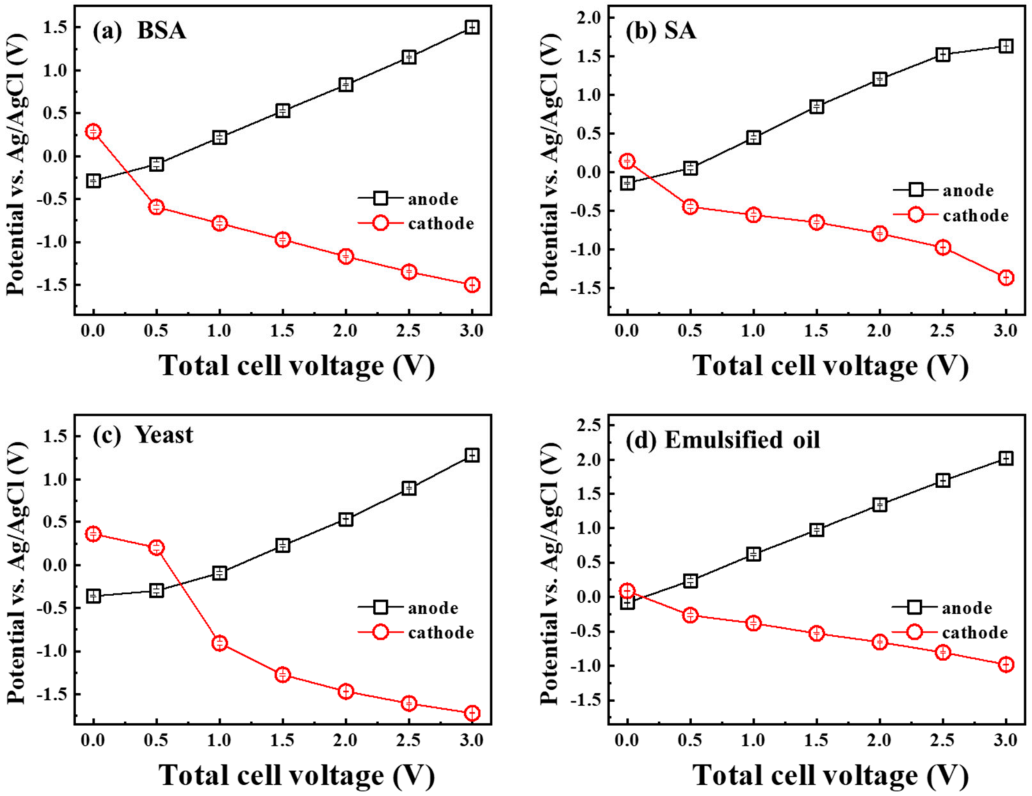

Figure S7. Anode/cathode potential distribution as a function of total cell potential: (**a**) BSA, (**b**) SA, (**c**) yeast, (**d**) emulsified oil. (Conditions: Car-PES memebrane cathode, titanium plate anode, and silver/silver chloride (Ag/AgCl) contrast electrode, $(BSA)_{in}$= 10 mg/L, $(SA)_{in}$= 10 mg/L,$(yeast)_{in}$= 10 mg/L, $(emulsified\ oil)_{in}$= 10 mg/L).

membranes

MDPI

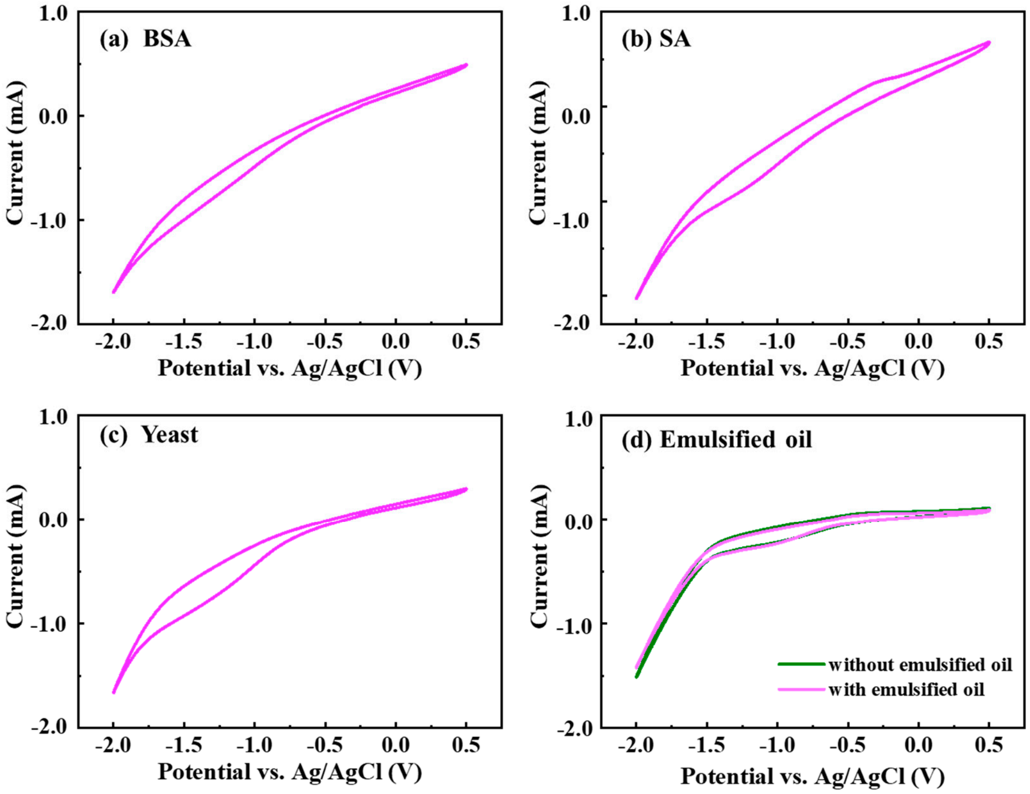

Figure S8. Cyclic voltammetry of the conductive Car-PES membrane with a scan rate of 100 mV·s^{-1}: (**a**) BSA, (**b**) SA, (**c**) yeast, (**d**) emulsified oil. (Conditions: Car-PES memebrane cathode, titanium plate anode, and silver/silver chloride (Ag/AgCl) contrast electrode, $(BSA)_{in}$= 10 mg/L, $(SA)_{in}$= 10 mg/L, $(yeast)_{in}$= 10 mg/L, $(emulsified\ oil)_{in}$= 10 mg/L).

Reference

1. Basri, H.; Ismail, A.F.; Aziz, M. Polyethersulfone (PES)–silver composite UF membrane: Effect of silver loading and PVP molecular weight on membrane morphology and antibacterial activity. *Desalination* **2011**, *273*, 72-80, doi:https://doi.org/10.1016/j.desal.2010.11.010.

© 2020 by the authors. Licensee MDPI, Basel, Switzerland. This article is an open access article distributed under the terms and conditions of the Creative Commons Attribution (CC BY) license (http://creativecommons.org/licenses/by/4.0/).
